# Noninvasive Diagnosis of Nonalcoholic Steatohepatitis and Advanced Liver Fibrosis Using Machine Learning Methods: Comparative Study With Existing Quantitative Risk Scores

**DOI:** 10.2196/36997

**Published:** 2022-06-06

**Authors:** Yonghui Wu, Xi Yang, Heather L Morris, Matthew J Gurka, Elizabeth A Shenkman, Kenneth Cusi, Fernando Bril, William T Donahoo

**Affiliations:** 1 Department of Health Outcomes & Biomedical Informatics College of Medicine University of Florida Gainesville, FL United States; 2 Target RWE Health Evidence Solutions Durham, NC United States; 3 Department of Medicine College of Medicine University of Florida Gainesville, FL United States; 4 Division of Endocrinology, Diabetes and Metabolism Department of Medicine University of Alabama at Birmingham Birmingham, AL United States

**Keywords:** machine learning, nonalcoholic fatty liver disease, nonalcoholic steatohepatitis, fatty liver, liver fibrosis

## Abstract

**Background:**

Nonalcoholic steatohepatitis (NASH), advanced fibrosis, and subsequent cirrhosis and hepatocellular carcinoma are becoming the most common etiology for liver failure and liver transplantation; however, they can only be diagnosed at these potentially reversible stages with a liver biopsy, which is associated with various complications and high expenses. Knowing the difference between the more benign isolated steatosis and the more severe NASH and cirrhosis informs the physician regarding the need for more aggressive management.

**Objective:**

We intend to explore the feasibility of using machine learning methods for noninvasive diagnosis of NASH and advanced liver fibrosis and compare machine learning methods with existing quantitative risk scores.

**Methods:**

We conducted a retrospective analysis of clinical data from a cohort of 492 patients with biopsy-proven nonalcoholic fatty liver disease (NAFLD), NASH, or advanced fibrosis. We systematically compared 5 widely used machine learning algorithms for the prediction of NAFLD, NASH, and fibrosis using 2 variable encoding strategies. Then, we compared the machine learning methods with 3 existing quantitative scores and identified the important features for prediction using the SHapley Additive exPlanations method.

**Results:**

The best machine learning method, gradient boosting (GB), achieved the best area under the curve scores of 0.9043, 0.8166, and 0.8360 for NAFLD, NASH, and advanced fibrosis, respectively. GB also outperformed 3 existing risk scores for fibrosis. Among the variables, alanine aminotransferase (ALT), triglyceride (TG), and BMI were the important risk factors for the prediction of NAFLD, whereas aspartate transaminase (AST), ALT, and TG were the important variables for the prediction of NASH, and AST, hyperglycemia (A_1c_), and high-density lipoprotein were the important variables for predicting advanced fibrosis.

**Conclusions:**

It is feasible to use machine learning methods for predicting NAFLD, NASH, and advanced fibrosis using routine clinical data, which potentially can be used to better identify patients who still need liver biopsy. Additionally, understanding the relative importance and differences in predictors could lead to improved understanding of the disease process as well as support for identifying novel treatment options.

## Introduction

Obesity, metabolic syndrome, and type 2 diabetes have reached epidemic proportions, and these conditions are strongly associated with nonalcoholic fatty liver disease (NAFLD) [1]. Consequently, NAFLD has become the most common type of chronic liver disease in both adults and children [2,3]. Data from the National Health and Nutrition Examination Survey showed that the prevalence of NAFLD has increased from 20% in 1988-1994 to 28.3% in 1999-2004 to 33% in 2009-2012 and leveled off at 32% in 2013-2016 [4]. Although NAFLD as well as nonalcoholic steatohepatitis (NASH) and fibrosis can be reversed in many cases with weight loss, these diseases remain significantly underdiagnosed; a recent electronic health record analysis of almost 18 million adults in Europe found the prevalence of NAFLD and NASH to be only 1.85% [5]. NAFLD ranges from isolated steatosis to NASH and cirrhosis. Knowing the difference between the more benign isolated steatosis and the more severe NASH and cirrhosis informs the physician regarding the need for more aggressive management. Unfortunately, these can only be distinguished through an invasive liver biopsy. As liver biopsies are associated with various complications and high expenses, there is an increasing interest in developing noninvasive methods to determine the stage of NAFLD [6].

Previous studies have explored several biomarkers as noninvasive surrogates, including markers of apoptosis [7], oxidative stress [8,9], and inflammation [10,11]. Several quantitative risk score calculators, such as the US Fatty Liver Index (US FLI) [12], aspartate aminotransferase-to-platelet ratio index (APRI) [13], and Fibrosis-4 (FIB-4) score [14], have been proposed and applied in clinical studies. These scores are easy and straightforward to calculate, yet they use data that are not routinely collected in the clinic (eg, the US FLI includes the waist circumference) or only use a limited number of variables (eg, APRI uses lab values for aspartate transaminase [AST] and platelets).

With the recent development of machine learning algorithms, we are now able to use clinical data in much more sophisticated ways. Perveen et al [15] applied a decision tree (DT) method to evaluate the risk of developing NAFLD in a Canadian population, where the onset of NAFLD is determined according to the clinical criteria, namely Adult Treatment Panel III. Islam et al [16] compared logistic regression (LR), random forests (RFs), and support vector machines (SVMs) for the prediction of fatty liver disease using gender, age, and 8 other variables from lab tests. Yip et al [17] compared LR, ridge regression, AdaBoost, and DT for NAFLD prediction using 6 predictors from routine clinical and laboratory variables.

Although machine learning methods have been applied to predict NAFLD, previous studies only focused on detecting NAFLD without discriminating between isolated steatosis and NASH, or advanced fibrosis. In addition, it is not clear how machine learning methods perform compared to existing quantitative calculators (eg, APRI) in predicting NASH or advanced fibrosis. Therefore, the aim of this project was to determine if machine learning algorithms could identify NASH or advanced liver fibrosis using commonly available clinical and biochemical data.

## Methods

### Data Set

Deidentified data from a NASH research database (KC) were used. Baseline data from a total of 492 participants who had been recruited from the general population as well as the hepatology and endocrinology clinics at the University of Florida in Gainesville, Florida, and the University of Texas Health Science Center at San Antonio in San Antonio, Texas, were included. Patients participating in this study were screened for NAFLD by routine chemistries and liver magnetic resonance spectroscopy. The final diagnoses of NASH and fibrosis staging were determined via a percutaneous liver biopsy. For collecting lab test data, the measurements were conducted at 1 point for each patient. All patients signed the informed consent form before participating in the study.

### Variable Encoding

To use the clinical and laboratory variables in machine learning algorithms, we compared 2 encoding methods including (1) categorical encoding, where the continuous lab values were converted into clinically meaningful categories according to domain experts; and (2) continuous encoding, where the continuous values were directly used without categorization. The categorical variables (eg, gender) were directly used in both encoding methods.

### Machine Learning Methods

We compared LR, DTs, RFs, SVMs, and gradient boosting (GB), 5 widely used machine learning algorithms, for the prediction of NAFLD, NASH, and advanced fibrosis. LR is a widely used statistical model that applies a logistic function to determine model dependency among variables. LR has been widely used in a number of clinical studies to assess associations or predict outcomes. In this study, we used LR as the baseline and compared it with other machine learning methods. DT and RFs are 2 tree-based machine learning methods that are widely used in data mining and machine learning. An SVM is a typical machine learning algorithm based on the large margin theory and has been applied to various prediction tasks. GB is a machine learning technology that produces a strong predictive model through ensembles of a number of weak models such as DTs. We implemented LR, DT, RFs and SVMs using the sciki-learn library [18] and implemented GB using the official XGBoost package.

### Feature Importance Analysis Using SHAP (SHapley Additive ExPlanations)

We also evaluated the important variables contributing to the prediction to examine how machine learning methods work using the SHAP method [19]. We used the feature importance, summary plot, and decision plot in SHAP to examine these variables. SHAP feature importance is a global importance score derived from the averaged absolute Shapley values per feature across the data set. Features with high SHAP importance are more influential for model prediction. The SHAP summary plot combines feature importance with feature effects. In a summary plot, each point is a Shapley value for a feature and an instance. The position on the y-axis is determined by the feature (ranked by the feature importance) and that on the x-axis by the Shapley value (positive or negative impact on model prediction). The color represents the feature value from low to high (red for high and blue for low). The summary plot is typically used to interpret the feature-model prediction association (positive or negative). The SHAP decision plot is used to show how features influence the models’ decision-making for individual samples. In a typical decision plot, there is a straight gray line indicating the model’s base value (starting point) and a colored line indicating prediction. Starting at the bottom of the plot, the prediction line shows how the SHAP values (ie, feature effects) accumulate from the base value to arrive at the model’s final score at the top of the plot. Thus, we can interpret which sets of features determine the model prediction results quantitatively. In this study, we adopted the decision plots for misclassification analysis.

### Existing NAFLD Risk Score Calculators

We examined 3 existing risk score calculators for the staging of liver fibrosis, including APRI [13] ([AST / 40] / platelets × 100), FIB-4 score [14] ([age × AST] / [platelets × √ALT]), and NAFLD fibrosis score (NFS) [20] (–1.675 + [0.037 × age] + [0.094 × BMI] + [1.13 × diabetes] + [0.99 × AST/ALT ratio] – [0.013 × platelets] – [0.66 × albumin]). We excluded the US FLI [12], as the waist circumference is not routinely measured in clinical practice.

### Experiments and Evaluation

For machine learning methods, we used 5-fold cross-validation and determined the area under the receiver operating characteristic curve (AUC or AUC-ROC) as the evaluation metric. In the 5-fold cross-validation, the 492 patients were divided into 5 equal groups. We trained the machine learning model using 5 groups and used the remaining group as the test set for prediction. We repeated this training/prediction procedure 5 times and shuffled the groups so that each group could get a chance to serve as the test set. The parameters of the machine learning methods were optimized according to the 5-fold cross-validation result (training curves shown in Figures S2 through S6 in Multimedia Appendix 1). Then, we calculated the specificity and sensitivity based on the Youden’s J statistic (Youden index) [21,22] determined from the ROC curve along with the AUC using the prediction from the 5-fold cross-validation. To reduce the bias of random grouping, for each machine learning method, we repeated the 5-fold cross-validation 20 times using different random seeds and calculated the mean specificity, mean sensitivity, mean AUC, and 95% CI. For existing scoring algorithms (APRI, FIB-4, and NFS), we used the bootstrapping strategy 100 times (80% data each time) to calculate the mean specificity, sensitivity, and AUC. Then, we selected the best machine learning method and compared it with existing scoring algorithms for the prediction of fibrosis. The mean AUC was used as the primary score for evaluation. All statistically significant parameters were identified by conducting 2-tailed *t* tests.

### Ethics Approval

This study was approved by the Institutional Review Board of the University of Florida (reference number: IRB201800923).

## Results

Baseline characteristics are presented in Table 1, separating patients based on the presence or absence of NASH. Tables S1and S2 (see Multimedia Appendix 1) present the baseline characteristics based on the presence or absence of advanced fibrosis and NAFLD, respectively.

Table 2 shows the performance of the machine learning methods for NAFLD prediction. The GB model with continuous encoding of variables achieved the best mean AUC score of 0.9043 (derived by performing the 5-fold cross-validation 20 times). The RF model with the continuous encoding method also achieved a comparable mean AUC score of 0.9020. Subsequent statistical analysis showed no significant difference (*P*=.61) between RFs and GB. Both GB and RFs outperformed the LR with *P*<.001 indicating statistical significance.

**Table 1:** Baseline characteristics of patients with and without nonalcoholic steatohepatitis (N=492).

Characteristic		Patients with NASH^a^ (n=198)	Patients without NASH (n=294)	*P* value^b^
Age, years, mean (SD)		55 ± 10	54 ± 11	.22
Males, n (%)		142 (72)	214 (73)	.88
**Ethnicity, n (%)**				<.001
	Caucasian	109 (55)	126 (43)	
	Hispanic	73 (37)	107 (36)	
	African American	11 (5.5)	55 (19)	
	Asian	3 (1.5)	4 (1)	
	Indian	0 (0)	2 (1)	
	Pacific Islander	2(1)	0(0)	
BMI, kg/m^2^, mean (SD)		34.1 (4.7)	33 (5.5)	.02
SBP^c^, mmHg, mean (SD)		134 (16)	134 (17)	.93
DBP^d^, mmHg, mean (SD)		79 (10)	78 (10)	.57
Total cholesterol, mg/dL, mean (SD)		183 (44)	168 (38)	<.001
TG^e^, mg/dL, mean (SD)		202 (148)	137 (85)	<.001
LDL-C^f^, mg/dL, mean (SD)		106 (36)	98 (34)	.03
HDL-C^g^, mg/dL, mean (SD)		39 (11)	43 (13)	<.001
A_1c_^h^, %		6.8 (1.3)	6.5 (1.2)	.004
AST^i^, IU^j^/L, mean (SD)		47 (26)	28 (14)	<.001
ALT^k^, IU/L, mean (SD)		64 (37)	37 (27)	<.001
Bilirubin, mg/dL, mean (SD)		0.9 (0.5)	0.8 (0.4)	.003
Platelets, 10^9^/L, mean (SD)		257 (84)	237 (63)	.006
Albumin, g/L, mean (SD)		4.2 (0.3)	4.1 (0.4)	.005
TSH^l^, mIU/L, mean (SD)		2.31 (1.51)	2.05 (2.41)	.14
FPG^m^, mg/dL, mean (SD)		136 (39)	127 (40)	.01
**Glucose** ** tolerance** ** (n, %)**				<.001
	Type 2 diabetes	144 (73)	181 (62)	
	Impaired glucose tolerance	41 (21)	48 (16)	
	Impaired fasting glucose	7 (3)	36 (12)	
	Normal glucose tolerance	6 (3)	29 (10)	
Presence of metabolic syndrome, n (%)		191 (96)	247 (84)	<.001
Presence of dyslipidemia, n (%)		180 (91)	206 (70)	<.001
Use of blood pressure medications, n (%)		159 (80)	181 (62)	<.001
Use of statins, n (%)		103 (52)	154 (52)	.99
Use of metformin, n (%)		92 (46)	119 (40)	.22
Use of sulfonylurea, n (%)		45 (23)	65 (22)	.96

^a^NASH: nonalcoholic steatohepatitis.

^b^For continuous variables, the *P* values were calculated by the 2-sided *t* test using 2 independent variables with unequal population variances. For categorical variables, the *P* values were calculated using the chi-square test.

^c^SBP: systolic blood pressure.

^d^DBP: diastolic blood pressure.

^e^TG: triglyceride.

^f^LDL-C: low-density lipoprotein-cholesterol.

^g^HDL-C: high-density lipoprotein-cholesterol.

^h^A_1c_: hyperglycemia

^i^AST: aspartate transaminase.

^j^IU: international units.

^k^ALT: alanine aminotransferase.

^l^TSH: thyroid-stimulating hormone.

^m^FPG: fasting plasma glucose.

**Table 2:** Performance of machine learning methods for prediction of nonalcoholic fatty liver disease.

Method and feature encoding		Mean sensitivity	Mean specificity	Mean AUC^a^ (95% CI)
**Logistic regression**				
	Categorical	0.7631	0.8557	0.8632 (0.8560-0.8704)
	Continuous	0.8232	0.8452	0.8786 (0.8716-0.8855)
**Support vector machines**				
	Categorical	0.8013	0.8112	0.8599 (0.8523-0.8676)
	Continuous	0.7773	0.8245	0.8524 (0.8455-0.8594)
**Decision tree**				
	Categorical	0.7297	0.7796	0.7932 (0.7835-0.8029)
	Continuous	0.7888	0.7809	0.8078 (0.7974-0.8183)
**Random forests**				
	Categorical	0.7811	0.8602	0.8782 (0.8717-0.8848)
	Continuous	0.8250	0.8595	0.9020 (0.8957-0.9083)
**Gradient boosting**				
	Categorical	0.7895	0.8380	0.8686 (0.8615-0.8756)
	Continuous	0.8343	0.8694	*0.9043 (0.8979-0.9107)*

^a^AUC: area under the receiver operating characteristic curve.

Table 3 compares the performance of the machine learning models in the prediction of NASH. The GB model with continuous encoding achieved the best mean AUC of 0.8166. The RF model with the continuous encoding method achieved a similar mean AUC score of 0.8119. Statistical comparisons between GB and RFs showed that *P*=.42, indicating no significant difference. Again, both GB and RFs significantly outperformed LR with *P*<.001 and *P*=.007, respectively.

Table 4 summarizes the performance of the machine learning methods in the prediction of advanced fibrosis. GB with the continuous encoding method achieved the best mean AUC of 0.8360. RFs with the continuous encoding method achieved a comparable mean AUC score of 0.8337, which is not significantly different from that of GB (*P*=.76). Although both GB and RFs outperformed LR in terms of the mean AUC score, subsequent statistical tests showed no significant difference between them (*P*=.29 between GB and LR; *P*=.46 between RFs and LR).

Next, we compared the best machine learning method (GB with continuous variable) with existing scoring algorithms in predicting advanced fibrosis. Table 5 shows the comparison results. The GB model outperformed the 3 existing scoring algorithms with an averaged AUC of 0.8360 for advanced fibrosis with significant *P* values. Among the 3 existing scoring algorithms, APRI achieved the best performance with an averaged AUC of 0.7890 in predicting the outcome. The AUC-ROC curves are provided in Figure S1 of Multimedia Appendix 1.

**Figure 1:**
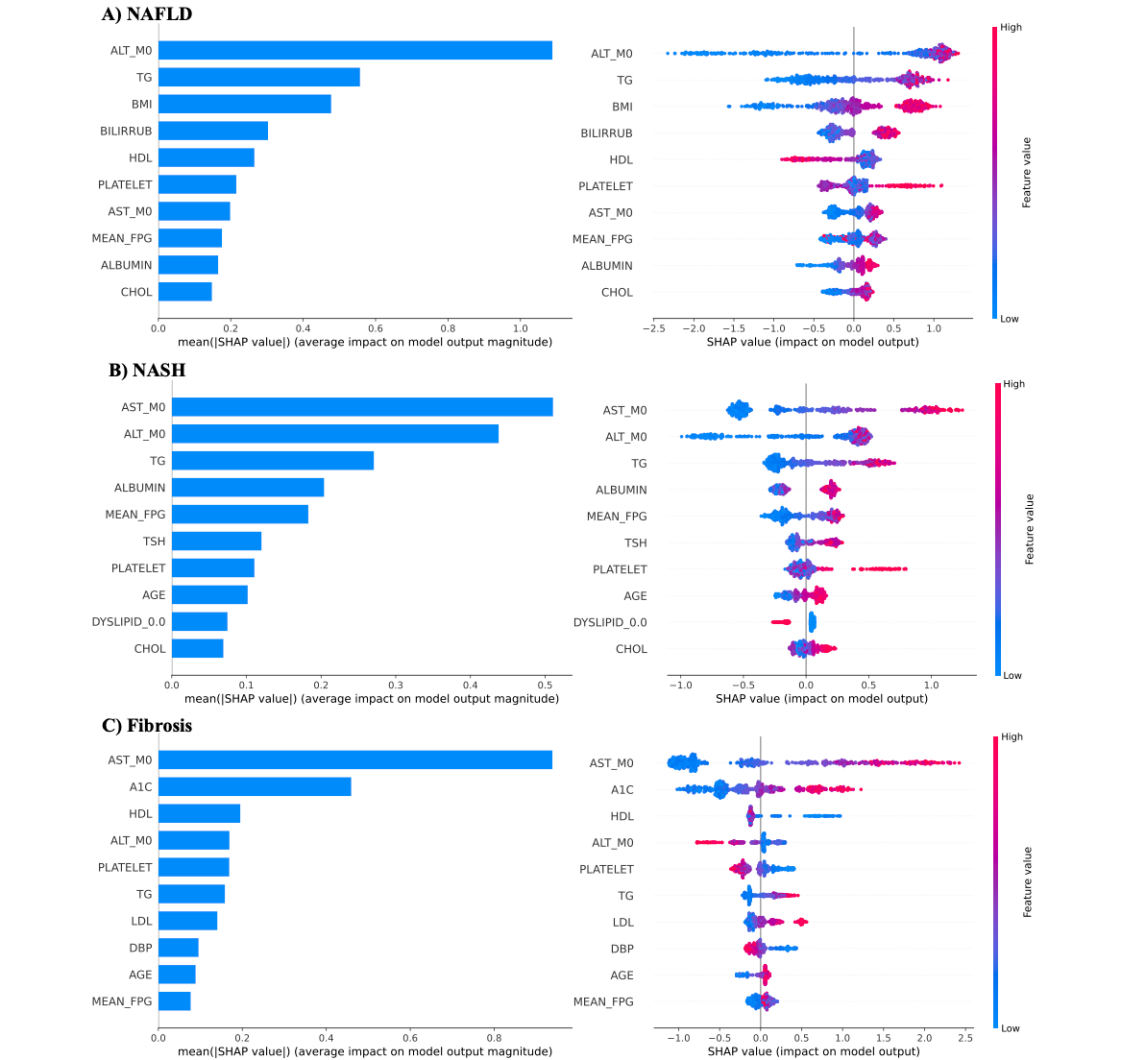
Top 10 important risk factors for prediction of NAFLD, NASH, and fibrosis based on SHAP importance calculated using the GB models with the continuous feature encoding method. (SHAP importance was derived from the averaged absolute SHAP values). A_1c_: hyperglycemia; ALT: alanine aminotransferase; AST: aspartate transaminase; BILIRRUB: bilirubin; CHOL: cholesterol; DBP: diastolic blood pressure; DYSLIPID: dyslipidemia; FPG: fasting plasma glucose; GB: gradient boosting; HDL: high-density lipoprotein; LDL: low-density lipoprotein; NAFLD: nonalcoholic fatty liver disease; NASH: nonalcoholic steatohepatitis; TG: triglyceride; TSH: thyroid-stimulating hormone; SHAP: SHapley Additive exPlanations.

Finally, we examined the importance scores of the top 10 variables for the disease states based on the SHAP values (see Table S2 in Multimedia Appendix 1). The top important variables for each condition were determined by the SHAP importance feature, which is defined as the mean absolution SHAP value. Figure 1 graphically demonstrates these results. For NAFLD, ALT was the most important variable (SHAP importance =1.02) followed by TG and BMI. For NASH, AST was the most important factor (SHAP importance=0.5) followed by ALT and TG. For advanced fibrosis, AST was the most important risk factor (SHAP importance=0.91) followed by hyperglycemia (A_1c_) and HDL.

**Table 3:** Performance of machine learning methods in prediction of nonalcoholic steatohepatitis.

Method and feature encoding		Mean sensitivity	Mean specificity	Mean AUC^a^ (95% CI)
**Logistic regression**				
	Categorical	0.7244	0.7523	0.7858 (0.7769-0.7948)
	Continuous	0.7070	0.7903	0.7956 (0.7871-0.8041)
**Support vector machines**				
	Categorical	0.7383	0.7480	0.7924 (0.7813-0.7983)
	Continuous	0.6836	0.8256	0.7968 (0.7886-0.8050)
**Decision trees**				
	Categorical	0.7064	0.6693	0.7201 (0.7098-0.7304)
	Continuous	0.6937	0.6881	0.7305 (0.7210-0.7401)
**Random forests**				
	Categorical	0.6979	0.8041	0.7910 (0.7819-0.8001)
	Continuous	0.7582	0.7691	0.8119 (0.8036-0.8215)
**Gradient boosting**				
	Categorical	0.7226	0.7600	0.7914 (0.7827-0.8001)
	Continuous	0.7525	0.7836	*0.8166 (0.8083-0.8249)*

^a^AUC: area under the receiver operating characteristic curve.

**Table 4:** Performance of machine learning methods in prediction of advanced fibrosis.

Method and feature encoding			Mean sensitivity		Mean specificity		Mean AUC^a^ (95% CI)
**Logistic regression**							
	Categorical	0.7683		0.7730		0.7950 (0.7837-0.8063)	
	Continuous	0.8500		0.7428		0.8278 (0.8172-0.8392)	
**Support vector machines**							
	Categorical	0.7367		0.7587		0.7628 (0.7489-0.7767)	
	Continuous	0.8242		0.7320		0.8122 (0.8002-0.8233)	
**Decision tree**							
	Categorical	0.7467		0.8010		0.7844 (0.7651-0.8037)	
	Continuous	0.6667		0.7379		0.6947 (0.6740-0.7153)	
**Random forests**							
	Categorical	0.7425		0.8529		0.8118 (0.7985-0.8251)	
	Continuous	0.8325		0.7757		0.8337 (0.8227-0.8447)	
**Gradient boosting**							
	Categorical	0.7492		0.8361		0.8115 (0.7977-0.8253)	
	Continuous	0.8083		0.8074		*0.8360 (0.8254-0.8467)*	

^a^AUC: area under the receiver operating characteristic curve.

**Table 5:** Comparison of gradient boosting (the best machine learning method) with existing scoring algorithms for prediction of advanced fibrosis^a^.

Method	Mean sensitivity	Mean specificity	Mean AUC^b^ (95% CI)	*P* value
GB^c^	0.8083	0.8074	0.8360 (0.8254-0.8467)	N/A^d^
APRI^e^	0.7424	0.7606	0.7984 (0.7964-0.8004)	<.001
FIB-4^f^	0.7176	0.6674	0.7394 (0.7371-0.7417)	<.001
NFS^g^	0.7506	0.5673	0.6843 (0.6777-0.6909)	<.001

^a^The scores for APRI, FIB-4, and NFS were calculated by bootstrapping 80% of the data from all 492 patients 100 times.

^b^AUC: area under the receiver operating characteristic curve.

^c^GB: gradient boosting.

^d^N/A: not applicable.

^e^APRI: aspartate aminotransferase-to-platelet ratio index.

^f^FIB-4: Fibrosis-4.

^g^NFS: Nonalcoholic Fatty Liver Disease Fibrosis Score.

## Discussion

### Principal Findings

In this study, we systematically compared 5 machine learning algorithms for prediction of NAFLD, NASH, and advanced fibrosis using variables from routine lab tests and patients’ demographics. We collected 33 variables from a total of 492 patients with NAFLD, NASH, and advanced fibrosis verified by liver biopsy. The experimental results show that the GB model achieved the best mean AUC scores of 0.9040, 0.8135, and 0.8360 for the prediction of NAFLD, NASH, and advanced fibrosis, respectively. This study demonstrated that it is feasible to use machine learning methods for noninvasive diagnosis of NAFLD, NASH, and advanced fibrosis.

We compared the best machine learning model, GB, with 3 existing risk score calculators (APRI, FIB-4, and NFS) and the comparison results showed that GB significantly outperformed the existing calculators in identifying fibrosis by leveraging more patient variables. Even though APRI is a simple calculator defined using only AST and Platelet, it achieved a decent performance in identifying fibrosis cases with a relatively small margin (~4%) compared to GB. Existing risk score calculators are defined using a limited number of variables; therefore, they are straightforward to calculate and easy to use in clinical settings. On the other hand, machine learning methods can achieve better performance by leveraging more variables from patients. The GB model significantly outperformed FIB-4 and NFS recommended in recent guidelines, indicating the potential use of machine learning models as screening tools for improved identification of advanced fibrosis in clinics.

To use the variables in machine learning methods, we compared 2 encoding methods including continuous encoding and categorical encoding. Categorical encoding used domain expert knowledge to categorize the continuous lab test values into different clinically meaningful categories (eg, low, normal, and high). In contrast, continuous encoding is purely a data-driven approach, using the lab values as they are and leaving the machine learning models to learn the cutoffs. The experimental results show that continuous encoding is better for representing lab values in machine learning methods.

To understand how the GB model predicts NAFLD, NASH, and advanced fibrosis, we examined the top 10 important features, as shown in Figure 1. For NAFLD (Figure 1A), the findings make clinical sense with ALT as the most important risk factor, followed by obesity (BMI) and an indirect measure of steatosis such as TG and HDL, which are inversely related to NAFLD in the SHAP summary plot (Figure 1A right). As expected, other risk factors were also positively associated with NAFLD. For example, a high ALT indicates a high probability of NAFLD. This is consistent with clinical practice. For NASH (Figure 1B), AST is the most important feature followed by ALT with a SHAP importance value comparable to that of AST, which is also consistent with clinical practice. However, when compared to NAFLD, we identified 3 novel features in the top 10, including atherogenic dyslipidemia (TG), hyperglycemia (fasting plasma glucose), and thyroid hormone status (thyroid-stimulating hormone). Abnormalities in the hepatic thyroid hormone metabolism are gaining momentum as conditions that may be linked with the development of steatohepatitis [23]. Similar to NAFLD, many features (Figure 1B right) have positive associations with NASH. As anticipated, AST was the most important feature for advanced fibrosis (Figure 1C); however, A_1c_ was a novel factor related to the development of advanced liver fibrosis and the second most important one. Some studies have suggested a link between A_1c_ and diabetes and NASH [24,25], but the relationship of diabetes with the severity of steatohepatitis and fibrosis remains controversial [26]. Their relevance can be best appreciated in the summary plot (Figure 1C right). The order of these variables only provides correlative evidence and certainly not cause and effect; however, data such as these can also lead to the generation of hypotheses pertaining to the relative role of adiposity vs insulin resistance vs hyperglycemia in the progression of liver disease from NAFLD to NASH, and then to advanced fibrosis, and offer insights into the opportunities for future targeted therapies.

**Figure 2:**
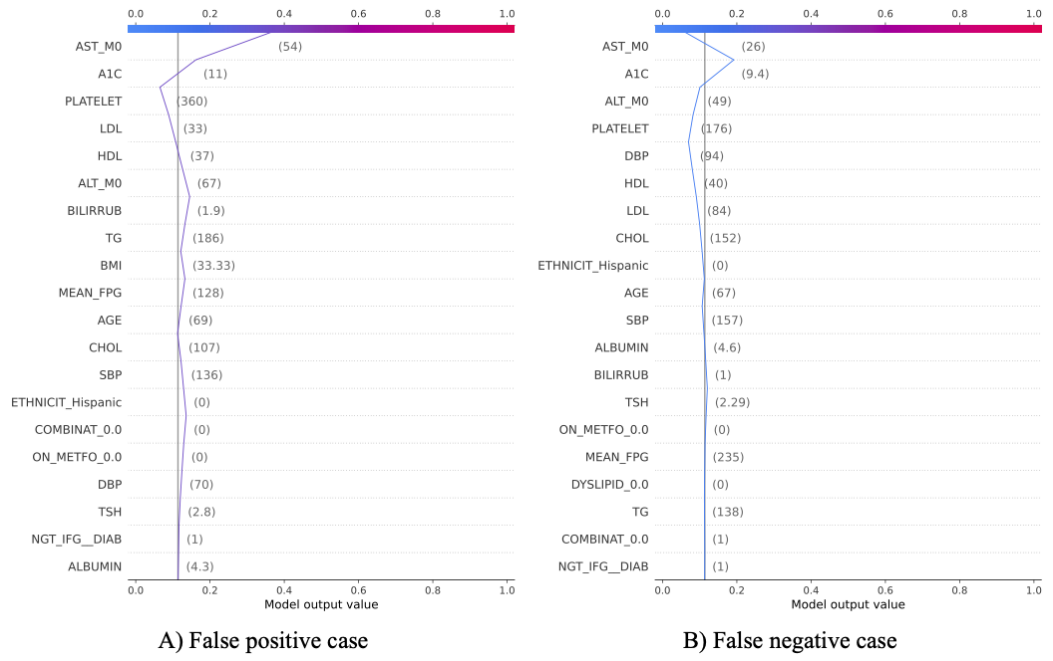
Decision plots for false positive and false negative prediction cases using the gradient boosting model with the continuous feature encoding method on advanced fibrosis. A_1c_: hyperglycemia; AST: aspartate transaminase; ALT: alanine aminotransferase; BILIRRUB: bilirubin; CHOL: cholesterol; DBP: diastolic blood pressure; DIAB: diabetes; DYSLIPID: dyslipidemia; FPG: fasting plasma glucose; HDL: high-density lipoprotein; IFG: impaired fasting glucose; LDL: low-density lipoprotein; METFO: metformin; NGT: narrow gastric tube; SBP: systolic blood pressure; TG: triglyceride; TSH: thyroid-stimulating hormone.

Figure 2 presents 2 error cases of the GB model in predicting advanced fibrosis. As for the false positive case (Figure 2A), this patient had no fibrosis according to the biopsy result (has NASH), but the model predicted fibrosis. The decision plot shows that the HDL (37 mg/dL), low-density lipoprotein (33 mg/dL), and Platelet (360K) of this patient are within the normal range, thus decreasing the SHAP value for fibrosis. However, the A_1c_ (11%) and AST (56 units per liter) of this patient are significantly higher than the normal range, which increased the SHAP value for fibrosis and led to the final predicted positive outcome. This observation is consistent with the feature importance analysis (Figure 1C) showing that A_1c_ and AST are strongly positively associated with the risk of advanced fibrosis. As for the false negative case (Figure 2B), the patient had advanced fibrosis determined from biopsy, but the GB model provided a negative prediction (no fibrosis). Although this patient has an A_1c_ of 9.4%, which increases the SHAP value, a normal AST (26 units per liter) significantly reduced the SHAP value and led to the final negative prediction outcome.

### Limitations

This study has limitations. First, the cohort in this study had 492 patients who were recruited at the University of Florida and the University of Texas Health Science Center. Future studies should examine our model using cohorts from different regions. Second, this study focused on 4 types or groups of medications as blood pressure medications, including statins, metformin, and sulfonylurea identified by the domain experts (physicians at the University of Florida). We plan to extend the data set and examine more medications (eg, obeticholic acid, pentoxifylline). Recent studies [27-30] showed that social determinants of health and environmental exposure are associated with the risk of liver diseases, which could be further explored.

### Conclusions

This study shows that it is feasible to use machine learning algorithms to identify NAFLD, NASH, and advanced fibrosis using common clinically available data. Further validation using larger and more clinically diverse data sets is required. Using only clinically available data, this method can effectively target individuals most likely to benefit from a liver biopsy to diagnose advanced liver disease. Additionally, understanding the relative importance of and differences in predictors could lead to improved understanding of the disease process and provide better support for identifying novel treatment options.

## Appendix

Multimedia Appendix 1 Supplementary tables and figures.

## Abbreviations

ALT: alanine aminotransferase
APRI: aspartate aminotransferase- to-platelet ratio index
AST: aspartate aminotransferase
AUC: area under the receiver operating characteristic curve
DT: decision tree
FIB-4: Fibrosis-4
GB: gradient boosting
HDL: high-density lipoprotein
LR: logistic regression
NAFLD: nonalcoholic fatty liver disease
NASH: nonalcoholic steatohepatitis
RF: random forest
ROC: receiver operating characteristic
SHAP: SHapley Additive exPlanations
SVM: support vector machine
TG: triglyceride
US FLI: US Fatty Liver Index
