# Supplementary material for: Noninvasive Diagnosis of Nonalcoholic Steatohepatitis and Advanced Liver Fibrosis Using Machine Learning Methods: Comparative Study With Existing Quantitative Risk Scores
Source: JMIR Med Inform. 2022 Jun 6;10(6):e36997. doi: 10.2196/36997 (PMC9210198; doi:10.2196/36997)
Supplement: Multimedia Appendix 1 [file medinform_v10i6e36997_app1.pdf]

Multimedia Appendix

This is a Multimedia Appendix to a full manuscript published in the J Med Internet Res. For full copyright and citation information see http://dx.doi.org/10.2196/jmir. 36997

Table S1. Baseline characteristics of patients with/without fibrosis.

	Patients with FIBROSIS (n=60)	Patients without FIBROSIS (n=432)	p-value [a]
Age, years	54 ± 11	56 ± 9	0.19
Gender, % of males	45 (75)	311 (72)	0.74
Ethnicity, n (%)			0.11
Caucasian	30 (50)	205 (47)	
Hispanic	28 (47)	151 (35)	
African-American	2 (3)	64 (15)	
Asian	0 (0)	10 (2)	
Indian	0 (0)	2 (0.5)	
Pacific Islander	0 (0)	2 (0.5)	
BMI, kg/m^2	33.2 ± 5.2	34.8 ± 4.9	0.03
SBP, mmHg	134 ± 17	132 ± 16	0.30
DBP, mmHg	79 ± 10	77 ± 10	0.18
Total cholesterol, mg/dl	173 ± 41	182 ± 45	0.17
TG, mg/dl	155 ± 110	222 ± 158	<0.01
LDL-C, mg/dl	101 ± 35	105 ± 36	0.33
HDL-C, mg/dl	42 ± 12	36 ± 9	<0.01
A1C, %	6.5 ± 1.2	7.5 ± 1.5	<0.01
AST, IU/L	33 ± 18	59 ± 33	<0.01
ALT, IU/L	44 ± 32	71 ± 40	<0.01
Bilirubin, mg/dl	0.8 ± 0.4	0.9 ± 0.6	0.23
Platelets, 10^9/L	247 ± 71	230 ± 83	0.14
Albumin, g/L	4.2 ± 0.4	4.1 ± 0.4	0.26
TSH, mIU/L	2.14 ± 2.19	2.23 ± 1.24	0.65
Fasting plasma glucose, mg/dl	129 ± 40	144 ± 40	<0.01
Glucose tolerance (n, %)			<0.01
Type 2 Diabetes	52 (87)	269 (62)	
Impaired Glucose Tolerance	5 (8)	88 (21)	
Impaired Fasting Glucose	3 (5)	40 (9)	
Normal Glucose Tolerance	0 (0)	35 (8)	
Presence of metabolic syndrome, n (%)	60 (100)	378 (88)	<0.01
Presence of dyslipidemia, n (%)	57 (95)	329 (76)	<0.01
Use of blood pressure medications, n (%)	50 (83)	290 (62)	0.02
Use of statins, n (%)	29 (48)	228 (53)	0.61
Use of metformin, n (%)	37 (62)	174 (40)	<0.01
Use of sulfonylurea, n (%)	20 (33)	90 (21)	0.04

Continuous variables were presented as "mean ± std"; categorical variables were presented as "number (percentage %)"

[a] For continuous variables, the p-values were calculated using the two-sided two independent samples T-test with unequal population variances. For categorical variables, the p-values were calculated using the Chi-square test.

Table S2. Baseline characteristics of patients with/without NAFLD

	Patients without NAFLD (n=146)	Patients with NAFLD (n=346)	p-value [a]
Age, years	56 ± 11	54 ± 10	0.07
Gender, % of males	95 (65)	261 (75)	0.03
Ethnicity, n (%)			<0.01
Caucasian	63 (43)	172 (50)	
Hispanic	36 (25)	143 (41)	
African-American	41 (28)	25 (7)	
Asian	4 (3)	3 (1)	
Indian	2 (1)	1 (0.3)	
Pacific Islander	0 (0)	2 (0.7)	
BMI, kg/m^2	31.5 ± 5.9	34.2 ± 4.6	<0.01
SBP, mmHg	136 ± 19	133 ± 15	0.12
DBP, mmHg	79 ± 11	78 ± 10	0.20
Total cholesterol, mg/dl	164 ± 34	178 ± 43	<0.01
TG, mg/dl	110 ± 66	185 ± 129	<0.01
LDL-C, mg/dl	95 ± 27	103 ± 38	<0.01
HDL-C, mg/dl	47 ± 13	39 ± 11	<0.01
A1C, %	6.4 ± 1.2	6.7 ± 1.3	0.02
AST, IU/L	24 ± 12	41 ± 23	<0.01
ALT, IU/L	25 ± 18	57 ± 35	<0.01
Bilirubin, mg/dl	0.8 ± 0.4	0.9 ± 0.4	<0.01
Platelets, 10^9/L	232 ± 54	251 ± 79	<0.01
Albumin, g/L	4.2 ± 0.4	4.2 ± 0.4	0.34
TSH, mIU/L	1.9 ± 1.4	2.3 ± 2.3	0.06
Fasting plasma glucose, mg/dl	128 ± 45	132 ± 38	0.35
Glucose tolerance (n, %)			<0.01
Type 2 Diabetes	88 (60)	237 (68)	
Impaired Glucose Tolerance	18 (12)	71 (21)	
Impaired Fasting Glucose	16 (11)	27 (8)	
Normal Glucose Tolerance	24 (17)	11 (3)	
Presence of metabolic syndrome, n (%)	115 (79)	323 (93)	<0.01
Presence of dyslipidemia, n (%)	95 (65)	291 (84)	<0.01
Use of blood pressure medications, n (%)	88 (60)	252 (73)	<0.01
Use of statins, n (%)	78 (53)	179 (52)	0.81
Use of metformin, n (%)	59 (40)	152 (44)	0.53
Use of sulfonylurea, n (%)	27 (18)	83 (24)	0.22

Continuous variables were presented as "mean ± std"; categorical variables were presented as "number (percentage %)"

[a] For continuous variables, the p-values were calculated using the two-sided two independent samples T-test with unequal population variances. For categorical variables, the p-values were calculated using the Chi-square test.

Parameter optimization for machine learning methods

For logistic regression, we optimized the optimization method (solver), the regularization parameter c, and the tolerance of termination criterion e. For Support Vector Machines, we used the Radial Basis Function (RBF) kernel and tuned the regularization parameter c, and the tolerance of termination criterion e. For Random Forests, we performed the optimization on the parameters including the number of trees (n_estimators), the tree splitting function, the tree max depth (max_depth). For Gradient Boosting, we optimized the learning rate (eta), the maximum depth of a tree (max_depth), and the number of boost trees (n_estimators) and chose the logistic regression for binary classification as the objective function to evaluate the training loss and regularization.

Computation Efficiency

The computational time varied from 1 min to 1 hour depending on the types of models and number of hyperparameters to search. The logistic regression and support vector machines (SVMs) model in general took less time (a few minutes for training) than ensemble tree-based methods such as XGBoost or Random Forests (about 1 hour). The time used for prediction on 492 patients is trivial (milliseconds).

The prediction time between empirical (i.e., APRI, FIB-4, and NAFLD fibrosis score) and machine learning models are comparable (in milliseconds). For example, the GB models used ~70 milliseconds to predict 492 patients whereas APRI used ~ 30 milliseconds.

Table S3. Mean SHAP values for all features used for prediction NAFLD, NASH, and advanced fibrosis based on the best machine learning model (XGBoost with features derived from the continuous feature encoding method).

**NAFLD**		**NASH**		**Fibrosis**	
**Feature name**	**Mean |SHAP Value|**	**Feature name**	**Mean |SHAP Value|**	**Feature name**	**Mean |SHAP Value|**
ALT_M0	1.0178	AST_M0	0.4994	AST_M0	0.9126
TG	0.5645	ALT_M0	0.3976	A1C	0.4545
BMI	0.4801	TG	0.2776	HDL	0.1956
BILIRRUB	0.3062	ALBUMIN	0.2099	PLATELET	0.1752
HDL	0.2814	MEAN_FPG	0.1838	ALT_M0	0.1698
PLATELET	0.2209	TSH	0.1224	TG	0.1565
AST_M0	0.1900	PLATELET	0.1197	LDL	0.1400
MEAN_FPG	0.1739	AGE	0.1057	DBP	0.0992
ALBUMIN	0.1716	DYSLIPID_0.0	0.0822	AGE	0.0930
CHOL	0.1462	ETHNICIT_Caucasian	0.0684	MEAN_FPG	0.0795
DBP	0.1340	CHOL	0.0681	TSH	0.0499
ETHNICIT_AA	0.1281	BILIRRUB	0.0601	BILIRRUB	0.0498
LDL	0.1232	BMI	0.0508	ETHNICIT_Hispanic	0.0497
A1C	0.1160	SBP	0.0457	BMI	0.0486
TSH	0.1135	ON_BP_ME_0.0	0.0420	CHOL	0.0449
ETHNICIT_Hispanic	0.1025	GNDR_0.0	0.0417	SBP	0.0342
SBP	0.0911	A1C	0.0408	ON_METFO_0.0	0.0326
AGE	0.0903	HDL	0.0313	ALBUMIN	0.0264
DYSLIPID_0.0	0.0496	COMBINAT_0.0	0.0146	DYSLIPID_0.0	0.0136
ON_METFO_0.0	0.0378	DBP	0.0131	NGT_IFG__DIAB	0.0129
ON_BP_ME_0.0	0.0342	ON_METFO_0.0	0.0107	COMBINAT_0.0	0.0106
ON_STATI_0.0	0.0332	ON_SU_0.0	0.0087	GNDR_0.0	0.0050
ETHNICIT_Caucasian	0.0272	LDL	0.0066	NGT_IFG__IFG	0.0000
NGT_IFG__IGT	0.0193	ETHNICIT_AA	0.0033	NGT_IFG__NGT	0.0000
ON_SU_0.0	0.0170	NGT_IFG__DIAB	0.0031	NGT_IFG__IGT	0.0000
NGT__PRE_PreDM	0.0161	NGT_IFG__IGT	0.0029	NGT__PRE_PreDM	0.0000
NGT_IFG__NGT	0.0138	ON_STATI_0.0	0.0028	NGT__PRE_NGT	0.0000
DYSLIPID_1.0	0.0128	ETHNICIT_Hispanic	0.0020	NGT__PRE_DIAB	0.0000
COMBINAT_0.0	0.0122	MS_0.0	0.0009	DM_STATU_0.0	0.0000
DM_STATU_0.0	0.0115	NGT_IFG__IFG	0.0000	DM_STATU_1.0	0.0000
NGT_IFG__IFG	0.0106	NGT_IFG__NGT	0.0000	MS_0.0	0.0000
ON_BP_ME_1.0	0.0092	NGT__PRE_PreDM	0.0000	MS_1.0	0.0000
ON_STATI_1.0	0.0065	NGT__PRE_NGT	0.0000	GNDR_1.0	0.0000
ON_SU_1.0	0.0060	NGT__PRE_DIAB	0.0000	ETHNICIT_PI	0.0000
DM_STATU_1.0	0.0035	DM_STATU_0.0	0.0000	ETHNICIT_Asian	0.0000

MS_0.0	0.0031	DM_STATU_1.0	0.0000	ETHNICIT_Indian	0.0000
COMBINAT_1.0	0.0031	MS_1.0	0.0000	ETHNICIT_Caucasian	0.0000
NGT__PRE_NGT	0.0022	GNDR_1.0	0.0000	ETHNICIT_AA	0.0000
GNDR_0.0	0.0020	ETHNICIT_PI	0.0000	ON_BP_ME_0.0	0.0000
ON_METFO_1.0	0.0017	ETHNICIT_Asian	0.0000	ON_BP_ME_1.0	0.0000
MS_1.0	0.0012	ETHNICIT_Indian	0.0000	DYSLIPID_1.0	0.0000
NGT_IFG__DIAB	0.0010	ON_BP_ME_1.0	0.0000	ON_STATI_0.0	0.0000
NGT__PRE_DIAB	0.0000	DYSLIPID_1.0	0.0000	ON_STATI_1.0	0.0000
GNDR_1.0	0.0000	ON_STATI_1.0	0.0000	COMBINAT_1.0	0.0000
ETHNICIT_PI	0.0000	COMBINAT_1.0	0.0000	ON_METFO_1.0	0.0000
ETHNICIT_Asian	0.0000	ON_METFO_1.0	0.0000	ON_SU_0.0	0.0000
ETHNICIT_Indian	0.0000	ON_SU_1.0	0.0000	ON_SU_1.0	0.0000

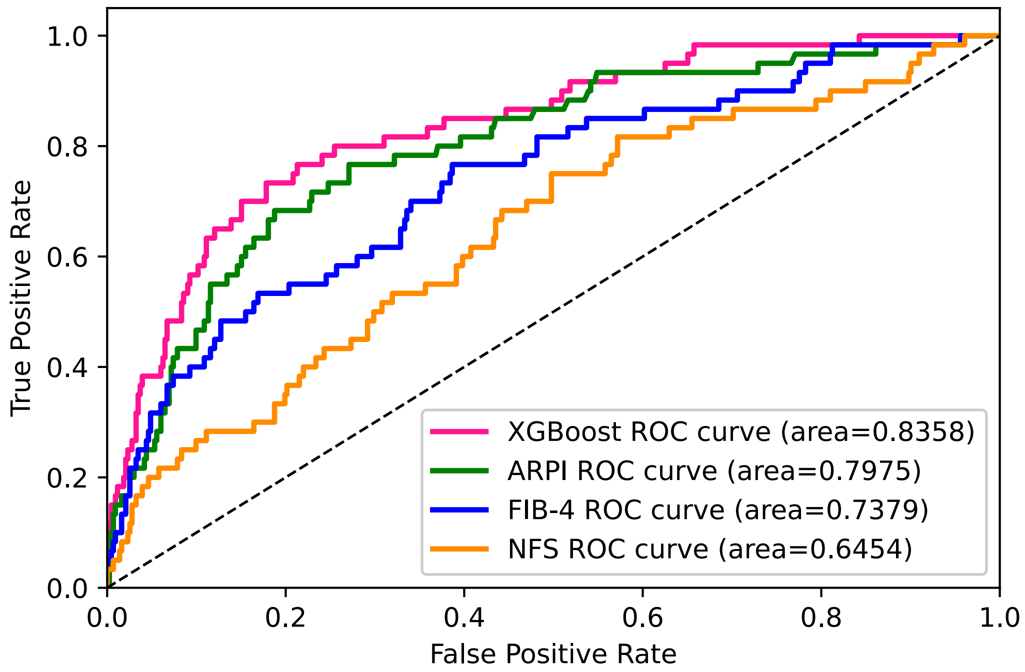

Figure S1. AUC-ROC Curves for measuring advanced fibrosis prediction performances for GB, APRI, FIB-4, and NFS, respectively. The AUC-ROC curve for GB was derived from a fivefold cross validation using random seed of 41. The AUC-ROC curves for APRI, FIB-4, and NFS were determined on all 492 patients without sampling.

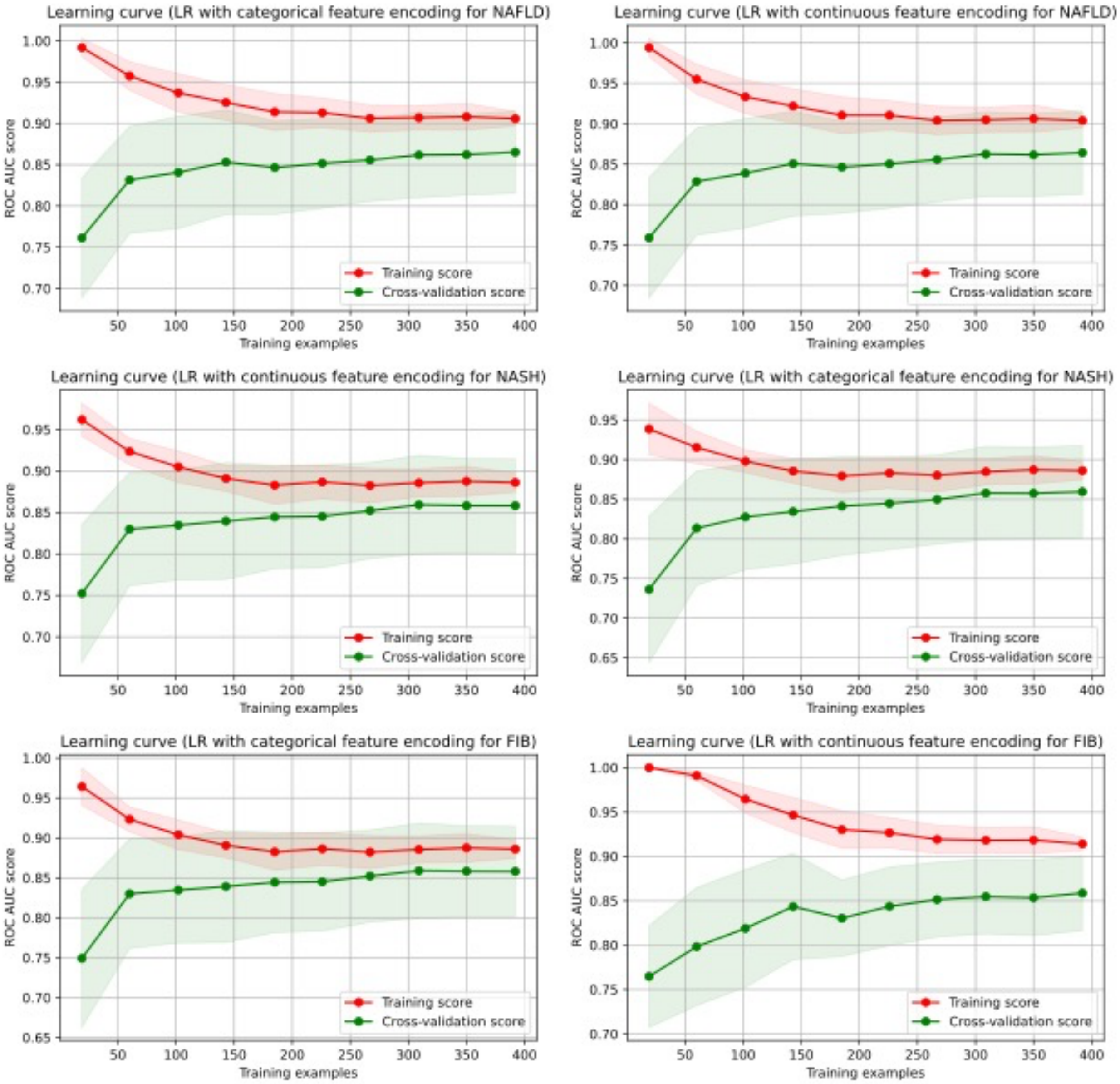

Figure S2. Learning curves for Logistic regression models.

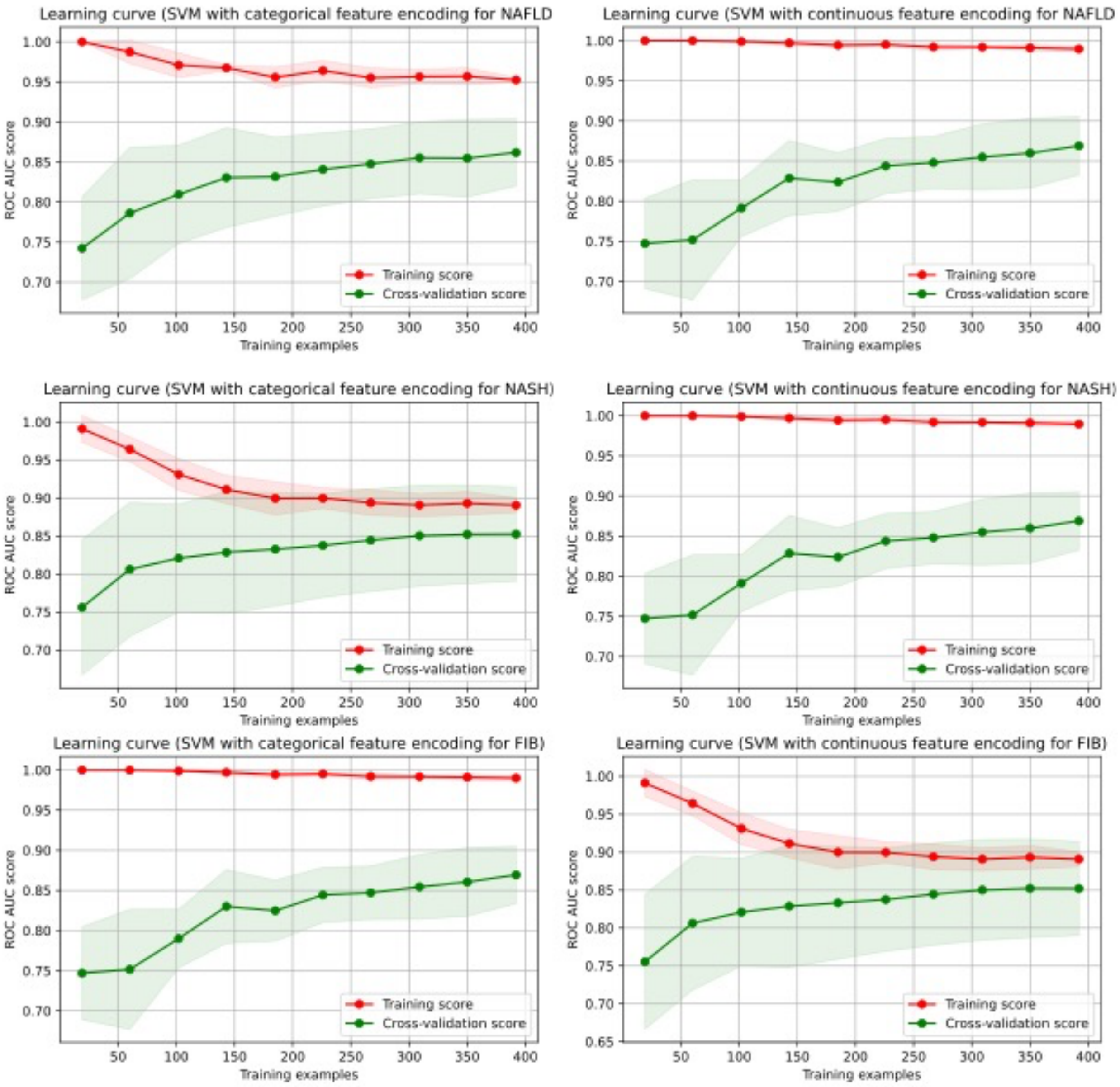

Figure S3. Learning curves for support vector machine models.

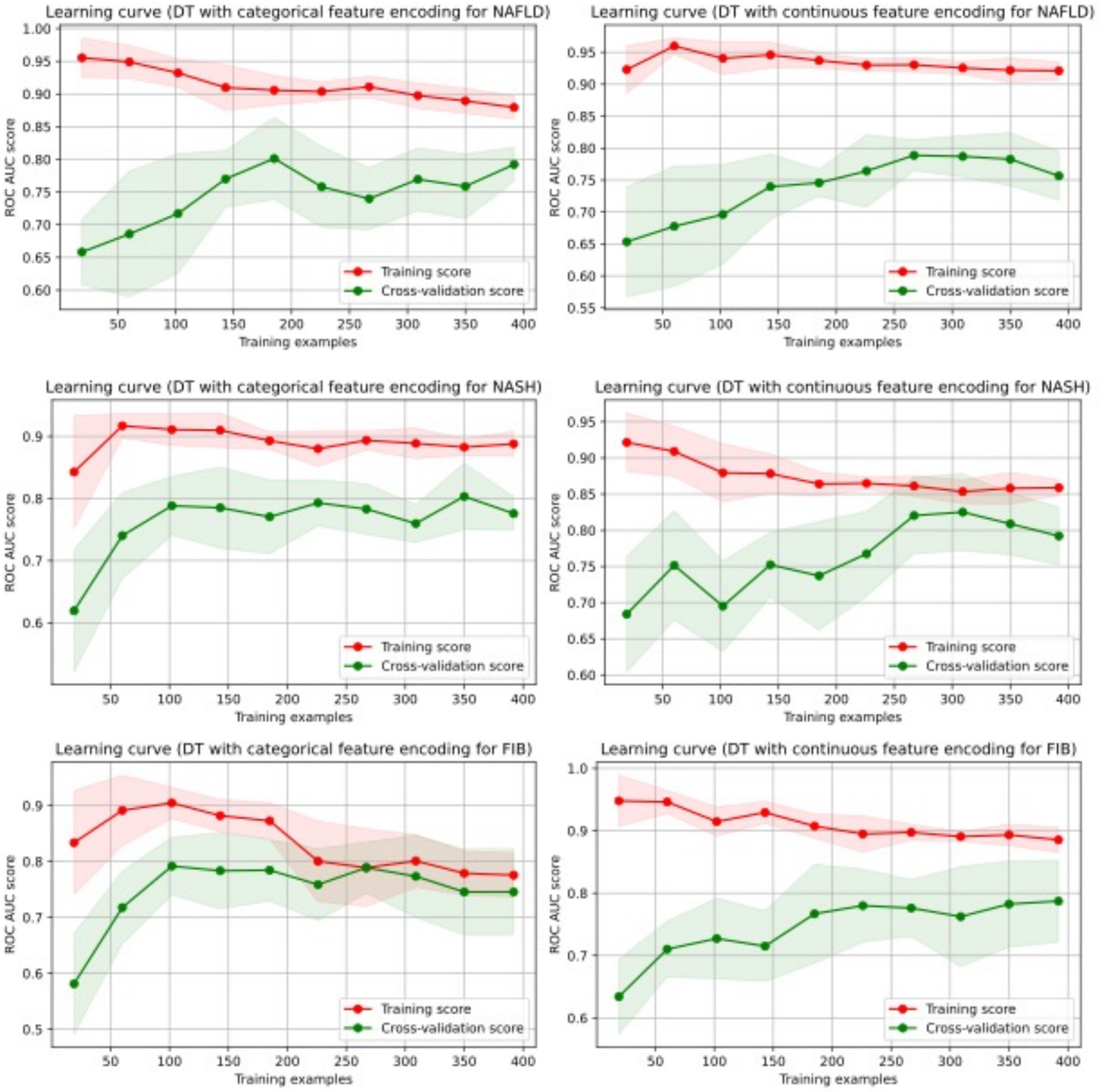

Figure S4. Learning curves for decision tree models.

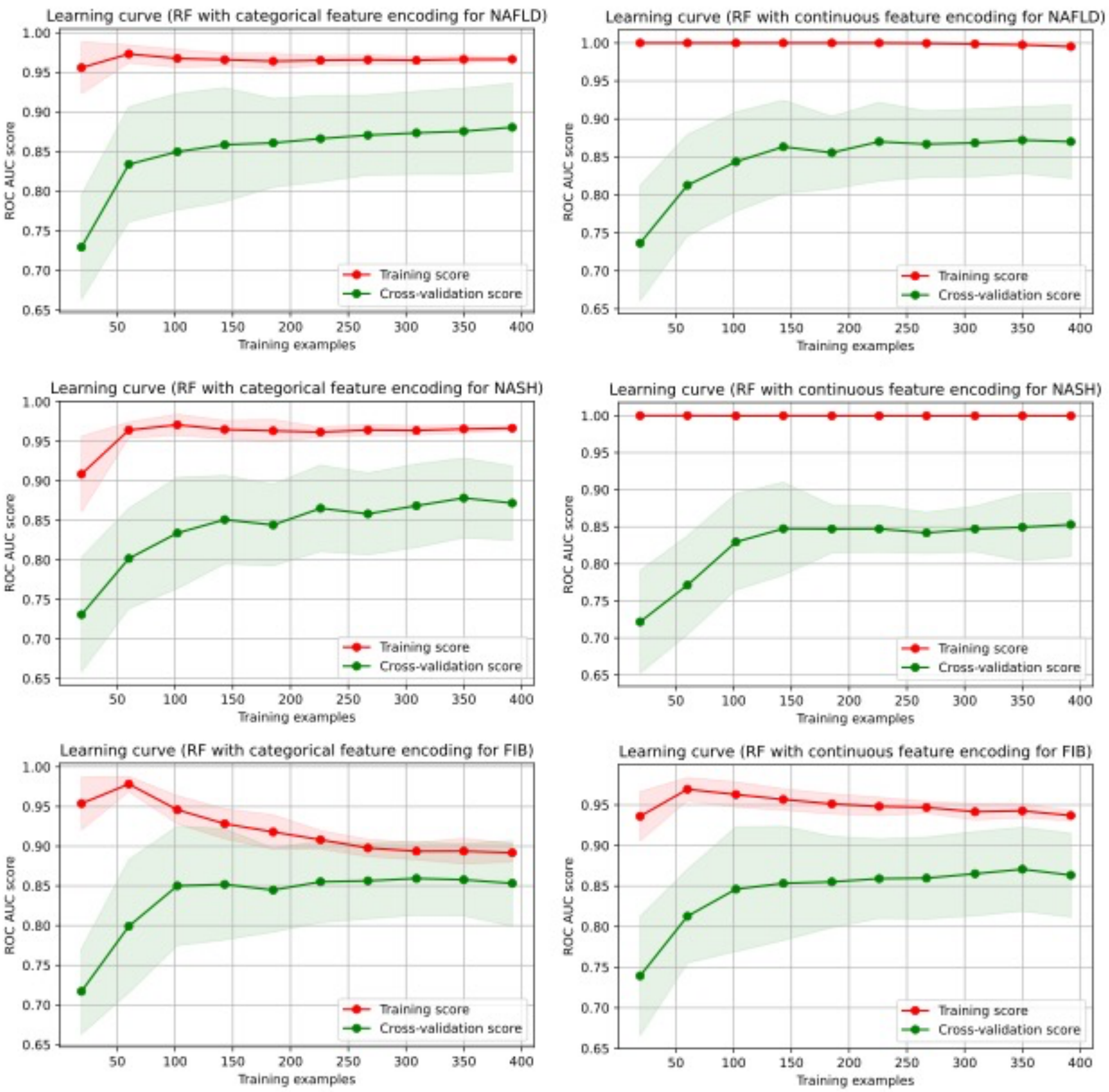

Figure S5. Learning curves for random forest models.

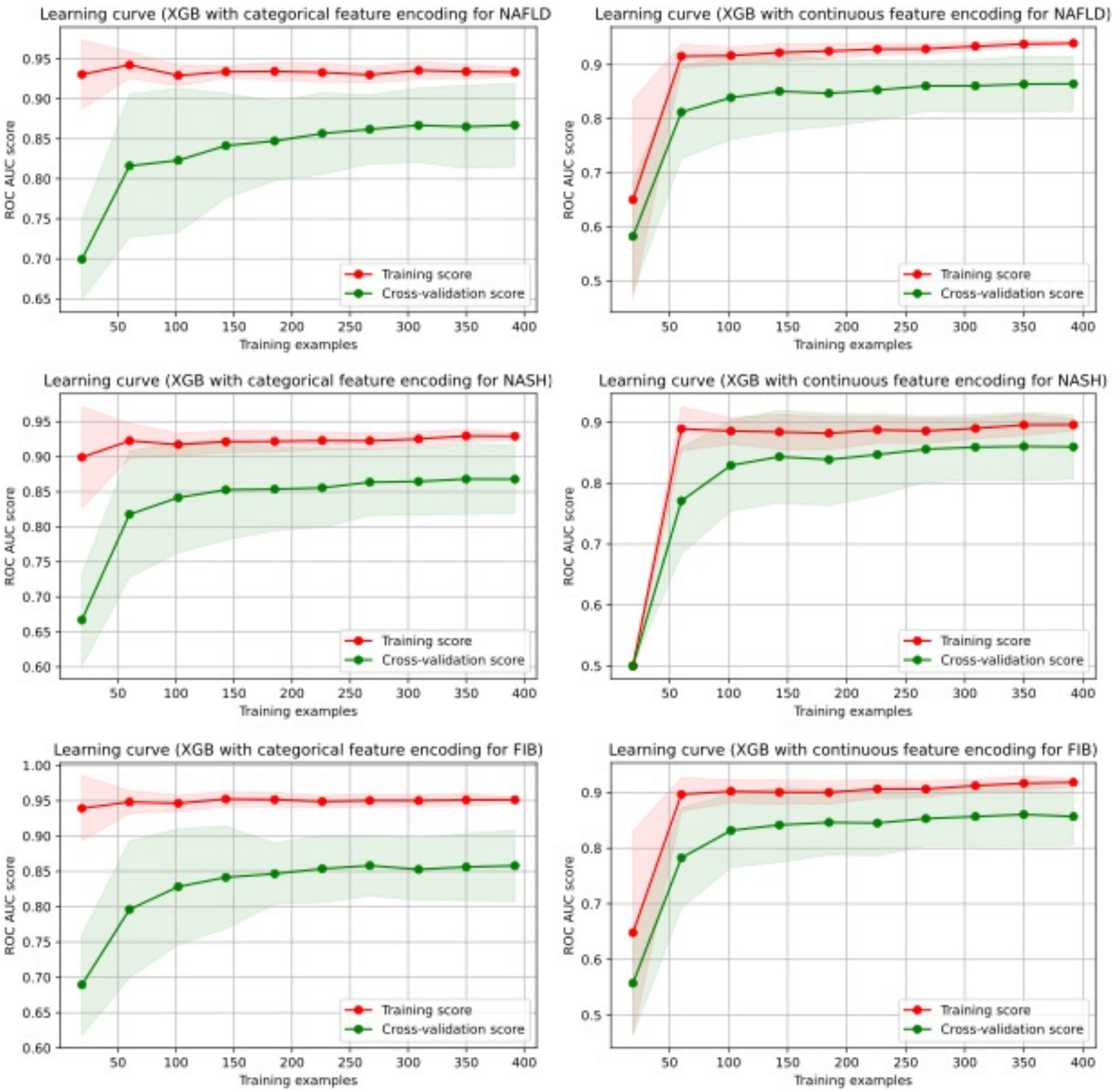

Figure S6. Learning curves for gradient boosting (XGBoost) models.
